# Rectal prolapse as the initial presentation of rectal cancer—A case report

**DOI:** 10.3389/fsurg.2023.1176726

**Published:** 2023-04-11

**Authors:** Oliver Jurić, Nataša Lisica Šikić, Vanja Žufić, Luka Matak, Robert Karlo, Jakov Mihanović

**Affiliations:** ^1^ Department of Surgery, Zadar General Hospital, Zadar, Croatia; ^2^ Department of Health Studies, University of Zadar, Zadar, Croatia; ^3^ Department of Pathology, Forensic Medicine and Cytology, Zadar General Hospital, Zadar, Croatia; ^4^ Department of Obstetrics and Gynecology, General Hospital Zadar, Zadar, Croatia

**Keywords:** rectal prolapse, rectal cancer, emergency department (ED), anemia, case report

## Abstract

Herein we report the case of a 63-year-old female tourist who presented to our Emergency Department with complete rectal prolapse. She had complained of diarrhea with traces of blood and mucus and had experienced fatigue after hiking. After the initial evaluation, it became clear that prolapse bares a large rectal tumor as a leading point. The prolapse was reduced under general anesthesia, along with a tumor biopsy. Further workup confirmed locally advanced adenocarcinoma of the rectum, which was treated with neoadjuvant chemoradiation followed by curative surgery in another hospital after repatriation. Rectal prolapse affects people of all ages, but it is more common in older adults, particularly women. Treatment options vary depending on the severity of the prolapse and can range from conservative measures to surgical interventions. This case report highlights the importance of early recognition and appropriate management of rectal prolapse in the emergency setting and the possibility of an underlying malignancy.

## 1. Introduction

Rectal prolapse or procidentia is a circumferential, full-thickness protrusion of the rectum through the anus. When irreducible and painful, it is classified as incarcerated (1). Delayed or unsuccessful reduction leads to strangulation, ischemia, bowel necrosis, perforation, and sepsis. Both conditions represent a complicated form of rectal prolapse mandating appropriate urgent treatment (2, 3).

Rectal prolapse is a rare condition that most commonly affects older adults, particularly women. The exact cause of rectal prolapse is unknown, but it has been associated with various factors, including chronic constipation, elongated rectosigmoid junction, weakened pelvic floor, and repetitive straining at defecation. Underlying bowel tumorous growth is a rare cause of prolapse but can occur even in children (4–6). A rising number of authors in the last decade published rectal or sigmoid adenocarcinoma cases leading to the presentation as a rectal prolapse (7–19).

Herein we report a 63-year-old female patient with the emergency presentation of incarcerated rectal prolapse caused by a locally advanced rectal tumor. The case is written in accordance with the CARE checklist (20).

## 2. Case description

A 63-year-old slim and fit female tourist arrived at the Emergency Department (ED) with complete rectal prolapse. A few days before, the patient had diarrhea with traces of blood and mucus. She recently noticed fatigue associated with intense hiking. After having the last stool in the mountains, a “massive prolapse” through the anus occurred. Ambulance Emergency Service transferred her to our hospital's ED. Until now, she didn't have any problems related to bowel habits and, therefore, never had an endoscopic workup. She took regular medication for hypertension.

A clinical examination revealed a complete rectal prolapse with a bulky exulcerated tumor occupying the posterior and right rectal wall, 8 cm × 10 cm in size (Supplementary Figure S1). The abdomen was soft and non-tender. The patient did not develop yet signs or symptoms of intestinal obstruction. There were no signs of hemodynamic instability. Her general condition was unimpaired, apart from the pale appearance of her skin.

## 3. Diagnostic assessment

Initial laboratory findings showed elevated white blood cell (WBC) count (26.8 × 10^9^/L; reference range 3.4–9.7), with predominant neutrophilia (87% of granulocytes; reference range 44%–72%), and almost normal C-reactive protein (CRP) level (6.4 mg/L; reference range 0–3). The complete blood count showed severe hypochromic microcytic anemia. Red blood cell (RBC) count was 3.41 × 10^12^/L (reference 4.34–5.72), hematocrit (HCT) was 21% (reference 41.5–53), hemoglobin (Hb) level was 62 g/L (reference 138–175), mean corpuscular volume (MCV) was 61.5 fl (reference 83–97.2), mean corpuscular hemoglobin (MCH) was 18.1 pg (reference 27.4–33.9), mean corpuscular hemoglobin concentration (MCHC) was 294 g/L (reference 320–345), with increased red cell distribution width (RDW) (18%; reference 9–15), and elevated platelets (PLT) count (528 × 10^9^/L; reference 158–424).

Since the patient was a foreign citizen, after explaining therapeutic options, a decision was made to reduce the prolapse and perform a tumor biopsy to complete the work-up after repatriation. Under general anesthesia, tumor samples were taken for biopsy, and the prolapse was easily reduced since the anal sphincter appeared significantly weakened. After the reduction, a further digit rectal examination was performed to assess the tumor's position and size. It was located on the posterior and right rectal wall, reaching almost the anal sphincter, albeit not infiltrating the sphincter itself.

The patient was kept in our department for three days, receiving three units of erythrocyte concentrate. Upon dismission, her laboratory findings showed improvement: WBC count dropped to 10.4 × 10^9^/L, whereas RBC increased to 4.47 × 10^12^/L, Hg to 93 g/L, and HCT to 31.8%. Besides, specific tumor markers showed elevated carcinoembryonic antigen (CEA) with 4.9 ng/ml (normal  < 4.3 ng/L), while the value of CA 19–9 marker was within normal limits (17.6 U/ml); reference value <39. The histopathology report confirmed that prolapsed rectal tumor was adenocarcinoma (Supplementary Figure S2). We emailed the pathology report to the patient and continued the correspondence, learning that she completed a workup followed by neoadjuvant chemoradiotherapy and abdominoperineal resection.

## 4. Discussion

The association between rectal prolapse and bowel tumorous growth is poorly represented in the literature. There is only one study, retrospective in design, published in 1995, convincingly demonstrating that the patients suffering from chronic rectal prolapse have a 4.2-fold higher relative risk for colorectal cancer than the representative cohort without rectal prolapse. The authors advocated routine screening for cancer using endoscopy, though no plausible explanation of the increased risk was offered (21). Most of the subsequent authors seconded their proposal for endoscopic surveillance based solely on sporadic case reports (7–11, 15–18). Speculation is that chronic straining, mechanical irritation of mucosa, and obstipation might increase the colorectal cancer risk in patients with chronic rectal prolapse. In favor of the claim that increased intraabdominal pressure precipitates prolapse of the rectal tumor, stands the case of a pregnant patient developing incarceration and strangulation of prolapsed rectum-bearing tumor amid delivery (12). Weakened or absent sphincter tone is certainly contributing factor, as demonstrated in a prolapse of rectal tumor years after Hartmann's procedure for fecal incontinence after obstetric sphincter injury (13).

A somewhat opposing opinion is offered by Akyuz et al., who don't find common ground for the etiology of rectal prolapse and the formation of malignancy, which is the process occurring independently, not assisted (18). The etiology is separate, although a new onset of rectal prolapse might indicate cancer due to bowel semi-obstruction and the patient's efforts in prolonged and vigorous straining (17, 18). This is especially true for younger patients, males, and for acute onset of prolapse—therefore, emergency presentation or atypical patient background are red flags that should urge the clinician to expand the diagnostic umbrella even more. It was true in our case, where the relatively healthy, physically active patient with the new onset of prolapse presented in the emergency setting.

In their comprehensive guideline on anorectal emergencies, the World Society of Emergency Surgery (WSES) and American Association for the Surgery of Trauma (AAST), issued in 2021, under the chapter on complicated rectal prolapse, authoritatively advise performing an urgent contrast abdominopelvic computed tomography scan in all patients except for those with hemodynamic instability (1). The intention is to detect complications such as bowel necrosis or perforation and not to miss the possibility of underlying bowel malignancy. The recommendation is based on low-quality evidence (level 2C—outcomes studies), focusing more on detecting complications of rectal incarceration than having a stronghold in available case reports of synchronous malignant tumors in prolapse. Nevertheless, detecting both complications and possible cancer is of utmost importance in deciding upon the best treatment for the patient (conservative vs. surgical) and tailoring the appropriate surgical approach (abdominal, perineal, laparoscopic vs. open).

There is a consensus that colorectal tumors can act as a leading point in the telescoping of the bowel in adults, being the cause of intussusception (22). The exact mechanism could be applied to sigmoid/rectal tumors that act as a leading point for invagination and subsequent tumor prolapse outside the anal canal. However, this remains ungrounded because more bowel tumors would present with rectal prolapse, and the published data do not support it. Namely, there are only a handful of published cases of rectal prolapse-containing cancer, with the first dating back to 2004 (8). It is possible that the condition was underreported because, in the last decade, there has been an increasing number of reports on this particular issue (8–19). We have researched published literature through PubMed using the keywords “rectal prolapse” and “rectal adenocarcinoma or cancer,” which yielded 75 articles. A detailed study of the articles and their references found only nine relevant cases of rectal cancer with rectal prolapse (8, 10, 12–17, 19), where two more reported prolapses of sigmoid cancer (9, 18).

Existing literature on rectal prolapse has focused chiefly on its diagnosis, treatment, and associated risk factors, with little attention paid to the possible association between rectal prolapse and colorectal cancer. In this case, the main challenge was identifying the rectal prolapse containing cancer in the emergency setting and resolving the imminent danger of rectal strangulation. The acute situation was translated to elective, allowing time to complete the work-up and repatriate our patient. This case report adds to the existing literature on rectal prolapse. It highlights the importance of early recognition and appropriate rectal prolapse management, especially with emergency presentation.

Clinicians, teachers, and researchers should be aware of the potential association between rectal prolapse and rectal cancer and consider this possibility when evaluating patients with rectal prolapse. Early diagnosis and prompt management can improve patient outcomes. Further research may be necessary to understand the relationship between rectal prolapse and rectal cancer and to develop more effective diagnostic and treatment strategies. Overall, it is crucial to maintain a high level of clinical suspicion for rectal cancer in patients with rectal prolapse to provide timely and appropriate care.

## Data Availability

The original contributions presented in the study are included in the article/**Supplementary Material**, further inquiries can be directed to the corresponding author.
