# Supplementary material for: Rectal prolapse as the initial presentation of rectal cancer—A case report
Source: Front Surg. 2023 Apr 11;10:1176726. doi: 10.3389/fsurg.2023.1176726 (PMC10126431; doi:10.3389/fsurg.2023.1176726)

Supplementary Material

Case report: Rectal prolapse as the initial presentation of rectal cancer

Oliver Jurić[1,2], Nataša Lisica Šikić[2,3], Vanja Žufić[1,2], Luka Matak[2,4], Robert Karlo[1,2], Jakov Mihanović[1,2*]

[1]Department of Surgery, Zadar General Hospital, Zadar, Croatia

[2]Department of Health Studies, University of Zadar, Zadar, Croatia

[3]Department of Pathology, Forensic Medicine and Cytology, Zadar General Hospital, Zadar, Croatia

[4]Department of Obstetrics and Gynecology, General Hospital Zadar, Zadar, Croatia

*** Correspondence:**
Jakov Mihanović MD PhD
mihanovic@gmail.com

1 Supplementary Figures

Supplementary Figure 1. Clinical presentation of incarcerated rectal prolapse with a large exulcerated posterior right rectal wall tumor.

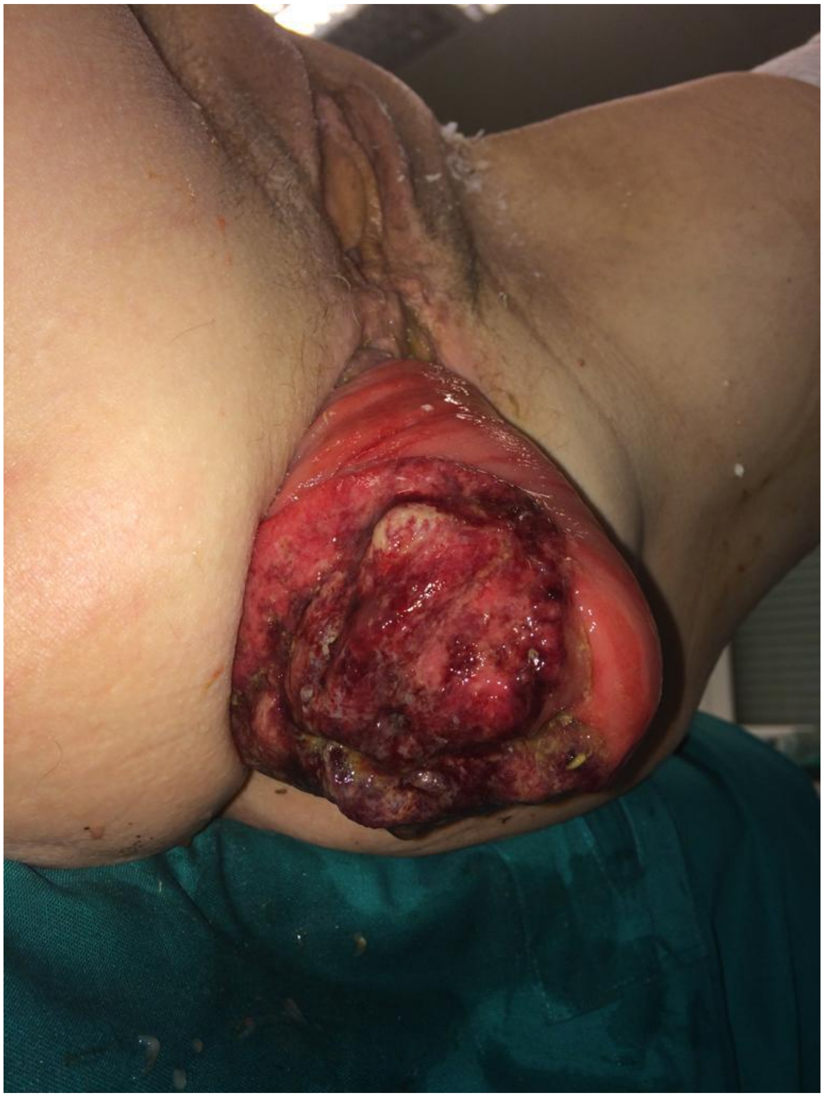

Supplementary Figure 2. Hematoxylin and Eosin stained specimen, 40x magnification light microscopy confirming adenocarcinoma of the rectum.

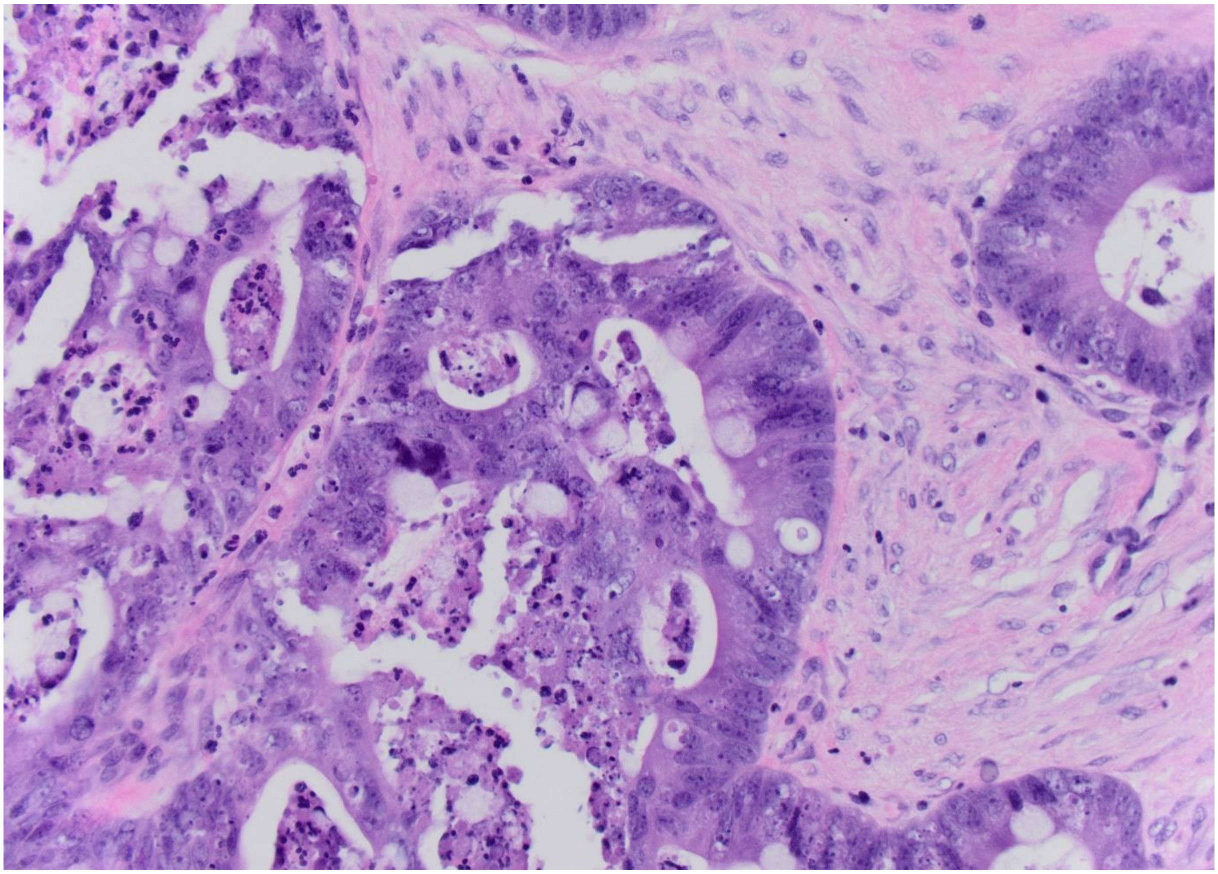

Supplement: Supplementary file 1 [file Datasheet1.pdf]
